# Airway Management for Penetrating Neck Trauma: A Case Report

**DOI:** 10.7759/cureus.33441

**Published:** 2023-01-06

**Authors:** João Oliveira, Nuno Maia, Joana Gonçalves, Valentina Almeida

**Affiliations:** 1 Anesthesiology, Centro Hospitalar e Universitário de Coimbra, Coimbra, PRT

**Keywords:** tracheal laceration, ­trauma, neck injury, endotracheal intubation, airway management

## Abstract

Penetrating neck injuries comprise 5-10% of traumatic injuries in adults and can cause immediate life-threatening compromise. Performing awake fibreoptic intubation in cooperative patients when airway management is not time critical has been suggested as a method of securing these potentially complicated airways. We report a case of a male in his 20s who presented to the emergency service with neck trauma following a bicycle road accident. With the exception of a wound in the neck region, there were no alarming distress signs or symptoms of airway endangerment. Imagiological evaluation revealed a rupture of the right lateral tracheal wall. He was referred for urgent surgery. We performed intubation with video laryngoscopy assisted by a neck surgery team, keeping the patient breathing spontaneously and under deep sedation. After advancing the tube through the vocal cords, the surgeon explored the cervical wound, guiding the tube through the trachea. Keeping spontaneous ventilation and advancing the tracheal tube beyond the lesion under visualization is essential when managing a traumatized airway. Tracheal intubation using video laryngoscopy, assisted by a neck surgeon guiding the tube, and avoiding creation of a false passage can be a safe alternative to fibreoptic intubation in selected cases of tracheal laceration.

## Introduction

Airway trauma is a potentially life-threatening condition that may be a result of blunt and penetrating injuries to the face, neck, and chest, as well as medical procedures that may injure the airway. The neck is an especially hazardous area for traumatic injuries. Penetrating neck injuries comprise 5-10% of traumatic injuries in adults [1]. Although countries like South Africa and other countries in Central and South America have the highest reported rates, countries in Europe have reported increasing levels of interpersonal violence over the last decade [2].

An anticipated incidence of 3-6% for cervical trachea injuries should be assigned to penetrating neck injuries [3]. Injuries in this area can be complex to manage, and airway management may be particularly perilous. Complications related to this injury can be catastrophic without optimal management. For example, in a patient with blunt or penetrating airway trauma, advancing a bougie or tracheal tube blindly beyond the vocal cords risks penetration through an airway laceration, leading to airway obstruction, pneumomediastinum, and the creation of a false passage [4].

In daily practice, anesthesiologists and intensivists may need to manage these potentially complicated airways. Performing awake fibreoptic intubation in cooperative patients when airway management is not time critical has been suggested as a method to secure these airways [5]. In this case report, we describe an alternative to fibreoptic intubation in airway management of a tracheal laceration after a penetrating neck injury.

## Case presentation

We report a case of a male patient in his 20s who presented to the ear, nose, and throat (ENT) emergency service showing neck trauma following a bicycle road accident. There were no signs of dyspnea and no other alarming distress signs or symptoms of airway endangerment. The patient complained only of mild localized pain in the traumatized neck region. Upon physical examination, a penetrating injury became apparent in the right anterior cervical region. A musculocutaneous tissue flap was identified over the injury. Behind it, an area of continuity between skin and tracheal lumen was found, where airflow could be heard during respiration and phonation. At palpation, small cervical subcutaneous emphysema was also apparent. Flexible nasendoscopy showed no laryngeal lesions and bilateral vocal cord movement was preserved. The patient was physically fit and had no other injuries and no previous medical history of any illness, surgeries, or allergies. Faced with a non-emergent airway hazard, it was decided to proceed with a careful imagiological evaluation prior to a surgical decision.

**Figure 1:**
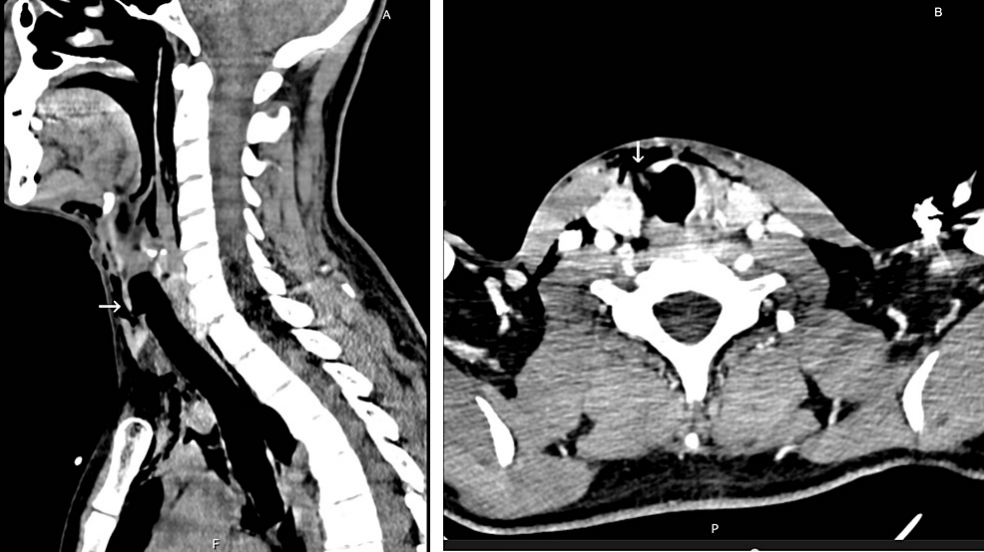
Sagittal computerized tomography scan showing tracheal laceration between the first and second tracheal rings (arrow) (A) and transverse imaging of the lesion (arrow) (B).

**Figure 2:**
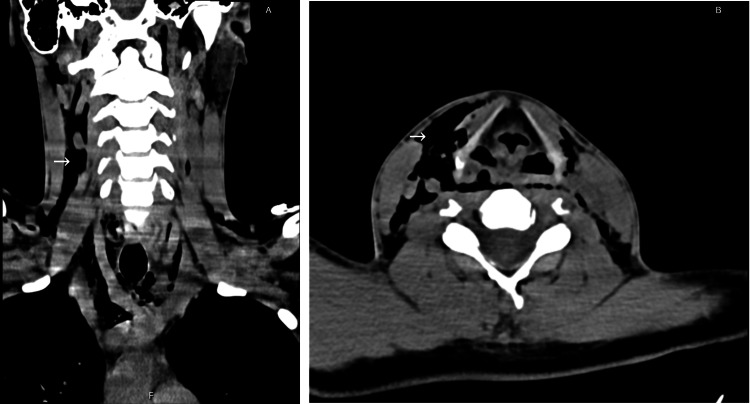
Cervical and subcutaneous emphysema (arrow) found in coronal computerized tomography (CT) scan (A) and transverse CT scan (B).

**Figure 3:**
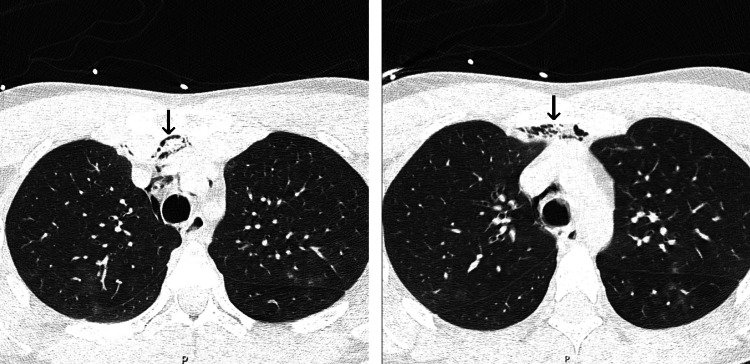
Pneumomediastinum (arrow) on computerized tomography scan.

A computerized tomography (CT) scan evaluation revealed a rupture of the right lateral tracheal wall between the first and second tracheal rings, next to the middle third of the right thyroid lobe (Figures 1A, 1B). The lesion was approximately 16 mm in length and presented with a flap of tissue over the injury. The skin lesion was cranially dislocated 10 mm from the tracheal lesion. As such, the area of continuity between skin and tracheal lumen was indirect. Additionally, the CT scan revealed cervical and subcutaneous emphysema as well as a small pneumomediastinum (Figures 2A, 2B, and Figure 3). Angiography phase showed no vascular damage.

The patient was then referred for uBrgent surgery. Operative planning was made after surgical and anesthetic team discussion. Two different possible strategies were defined - conventional orotracheal intubation followed by surgical correction or upfront tracheotomy with postponed surgical correction. Post-anesthetic care unit availability was assured.

Other than recent trauma, the patient had no other risk factors for airway aspiration. Still, adequate fasting time was assured and intravenous metoclopramide prokinetic premedication was administered before airway approach. Conventional airway assessment revealed no further signs of airway difficulty. Difficult airway equipment was present at all times in the operating room. Standard American Society of Anesthesiology monitoring was used as well as invasive arterial pressure catheter and anesthetic depth monitoring with bispectral index (BIS^©^; Minneapolis, MN: Medtronic). Preoxygenation was carefully done by facemask with no positive pressure applied to ensure no further worsening of the injury.

**Figure 4:**
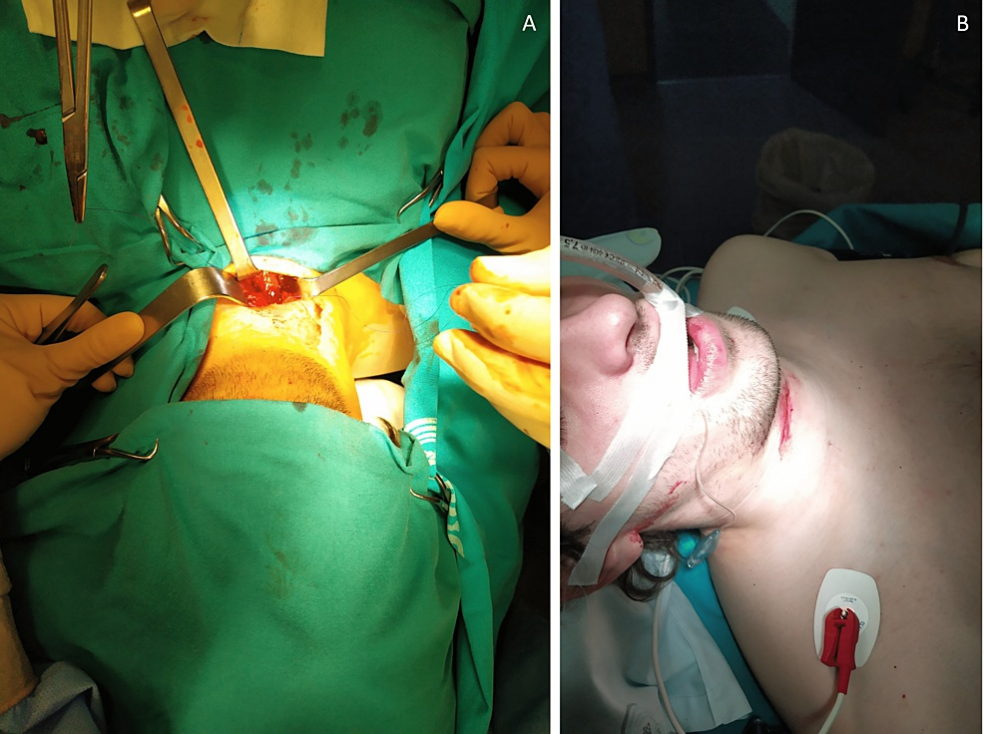
Surgical exploration of tracheal laceration (A) and immediate post-operative status (B).

Intravenous induction with propofol was carefully titrated in small fractions aiming to maintain spontaneous ventilation during airway approach. Laryngoscopy was performed using a video laryngoscope and an orotracheal tube was passed through the glottis. Then, the ENT team explored the cervical injury and helped guide the orotracheal tube down the trachea and past the laceration, avoiding further damage. Close communication and cooperation between medical teams were needed. Once the airway was secured, neuromuscular blockade was administered and surgery to repair the tracheal laceration proceeded (Figure 4A). Anesthetic depth was maintained with sevoflurane. Surgery was uneventful and lasted approximately one hour (Figure 4B). A nasogastric tube was placed at the end of the procedure for post-operative feeding.

After surgery, the patient remained intubated and sedated and was transported to the post-anesthetic care unit (PACU) for airway protection under strict surveillance, mechanical ventilation, and antiedematous therapeutics. At 48 hours of PACU stay, sedation was stopped, and the patient was extubated without complications. Extubation was done in the presence of ENT surgeons, in case an emergency tracheostomy was needed.

Corticotherapy and prophylactic antibiotics were instituted for prevention of further airway complications at the ENT ward. In order to detect lesion leakage, a methylene blue sample was given to the patient orally and no bronchial aspiration was detected. Post-operative flexible nasendoscopy detected only slightly diminished mobility of the right vocal cord. The patient was discharged home on day five of hospital stay with no complaints other than a small dysphonia, which disappeared after three months. An anesthetic warning document was placed in the hospital records, advising of possible future airway difficulty. After six months of medical follow-up, there was no dyspnea, dysphonia, or dysphagia. Flexible nasendoscopy revealed no other complications.

## Discussion

Due to the abundance of vital structures within the neck region, the risk of immediate life-threatening events is substantial after trauma to the airway. In fact, upon surgical exploration, some vascular or aerodigestive injuries can be disguised under a clinically superficial stab wound [6,7]. The assessment of neck wounds can be effectively achieved using the methodology first described by Monson et al. in 1969, which marks anatomical zones of injury by dividing structures of the head and neck [8]. The area from the clavicles to the cricoid cartilage is defined as zone 1, the area from the cricoid cartilage to the angle of the mandible is zone 2, and the area from the angle of the mandible to the base of the skull is zone 3. This topographical division helps in anticipating potential injuries and possible need for urgent airway management. Due to the abundance of vascular structures and proximity to the thoracic cavity of zone 1, the wounds presenting in this area have the highest associated mortality [9]. Wounds in the anterior and lateral aspects of the neck compromise the airway more often than those in the posterior region.

Common presenting signs and symptoms, such as cough, dyspnoea, aphonia, stridor, laryngeal crepitus, hemoptysis, and subcutaneous emphysema are non-specific. Also, there is no strong correlation between anatomical site of lesion and clinical presentation. However, the severity of injury is expected to be higher if a patient complains of hemoptysis and stridor at presentation [9]. Our patient presented with aphonia and a lateral cervical open wound in zone 1 with no sign of a compromised airway. He was submitted to a CT scan to identify the extent and location of airway injury; since it is the recommended initial work-up investigation in patients showing penetrating neck injuries and clinical stability [9]. Previous planning of airway management is essential and can be aided by routine airway assessment as well as cervical CT. The specificities of neck trauma render some elements of previously published national bodies' guidelines on airway management inappropriate or non-applicable [10,11].

The literature available in the field of airway management of a traumatized airway shows that a rapid sequence induction with the Sellick maneuver should be avoided. The use of cricoid pressure can displace laryngotracheal fractures and distort the airway anatomy, worsening the difficulty of laryngoscopy. It may also precipitate or aggravate airway bleeding, and its primary objective of esophagus compression may not even be achieved effectively. There is also a considerable risk of producing an air leak through airway traumatic wounds if positive pressure ventilation is applied via face mask or supraglottic device. Accumulation of air in the tissues may produce progressively greater surgical emphysema which can ultimately produce airway compromise. Anesthetic induction with some intravenous hypnotics and neuromuscular relaxants presents additional risk of apnea and complete collapse of airway, which could previously be deceptively considered safe due to the muscle tone of surrounding neck structures [3,9]. Therefore, maintenance of spontaneous breathing should be sought until securing the airway. Blindly placing a tracheal tube or bougie distal to the vocal cords can create a false passage by accidentally dislodging damaged tissues and such devices may even exit the trachea through an anatomical defect caused by the trauma. If a false tract is posteriorly submitted to positive ventilatory pressure the neck anatomy can be perilously distorted with possible complete obstruction of the airway [9]. Thus, visualization of the anatomy until the tracheal tube is distal to the level of breach is recommended. Finally, conventional emergency front-of-neck access via cricothyroidotomy is not recommended as it will worsen any anatomical distortions already in place in a scenario of laryngotracheal injury [5].

Performing awake fibreoptic intubation in cooperative patients when airway management is not time critical is the conventional approach, based on the recommendations of the Canadian Airway Focus Group for management of the difficult airway. These updated recommendations are based on a review of the anesthetic management of non-iatrogenic acute adult airway trauma [5,12]. Although awake fibreoptic intubation in skilled hands has proved effective, its execution can be quite difficult in the presence of blood or debris in airway [9]. Furthermore, in our scenario having direct airway access and visualization through the cervical wound largely mitigated any benefits that awake fibreoptic intubation would otherwise provide.

The management of complex anticipated airway difficulties resorts largely on human factors. Recently published guidelines by national bodies on airway management devote a significant section to these [11-13]. Airway security frequently falls back on vital non-technical aspects of medical practice, such as communication, leadership, and a shared mental model.

We consider our airway management plan a safe alternative to fibreoptic intubation in a patient with an open cervical wound that has communication with the trachea, when there is adequate time for airway assessment and investigation before airway management and no other airway injury is present. Our patient had adequate fasting time. The only aspiration risk factor was recent trauma, and he was therefore categorized as having intermediate risk [14]. Prokinetic medication was previously administered to reduce that risk. Before starting airway management, all the equipment needed was available and all team members were aware of the intubation plan. During our approach we kept patient’s spontaneous ventilation avoiding intravenous induction, neuromuscular blockade, and positive pressure ventilation via a face mask. We advanced the tracheal tube beyond the lesion under direct visualization through the wound to prevent creating a false passage. This was done under deep sedation to decrease patient stress during the procedure and to ensure adequate conditions for surgical exploration of the cervical wound. In the case of failure of the primary plan, our rescue plan was the insertion of a tracheostomy tube through the wound, as has already been reported in the literature [15].

## Conclusions

In conclusion, we describe the successful management of a tracheal laceration without acute respiratory distress. Keeping spontaneous ventilation and advancing the tracheal tube beyond the lesion under visualization is essential when managing a traumatized airway. Tracheal intubation using video laryngoscopy, assisted by a neck surgeon guiding the tube and avoiding creation of a false passage can be a safe alternative to fibreoptic intubation in selected cases of tracheal laceration.
